# Supplementary material for: Precise and stable edge orientation signaling by human first-order tactile neurons
Source: eLife. 2022 Oct 31;11:e81476. doi: 10.7554/eLife.81476 (PMC9642991; doi:10.7554/eLife.81476)
Supplement: Peer review history

# Editor report: Editor's evaluation

**DOI:** 10.7554/eLife.81476.sa0

**Authors:** Hannes P Saal

**Affiliations:** https://ror.org/05krs5044 University of Sheffield United Kingdom

This paper will be of broad interest to anyone aiming to understand the neural basis of human touch perception. This is an important paper that provides compelling evidence for peripheral tactile encoding of orientation that reflects perceptual capabilities, by using a wide range of stimulus conditions. The results will be valuable to inform both future experiments and computational investigations into the neural representation and processing of small tactile spatial features at the edge of perceptual resolvability and on the emergence of invariant representations in touch more generally.

# Decision letter

**DOI:** 10.7554/eLife.81476.sa1

**Affiliations:** https://ror.org/05krs5044 University of Sheffield United Kingdom; https://ror.org/05krs5044 University of Sheffield United Kingdom; https://ror.org/02495e989 Université Catholique de Louvain Belgium; https://ror.org/05ynxx418 Linköping University Sweden

Our editorial process produces two outputs: (i) public reviews designed to be posted alongside the preprint for the benefit of readers; (ii) feedback on the manuscript for the authors, including requests for revisions, shown below. We also include an acceptance summary that explains what the editors found interesting or important about the work.

**Decision letter after peer review:**

Thank you for submitting your article "Precise and stable edge orientation signaling by human first-order tactile neurons" for consideration by *eLife*. Your article has been reviewed by 3 peer reviewers, including Hannes P Saal as Reviewing Editor and Reviewer #1, and the evaluation has been overseen by Tamar Makin as the Senior Editor. The following individuals involved in review of your submission have agreed to reveal their identity: Benoit Delhaye (Reviewer #2); Saad S Nagi (Reviewer #3).

The reviewers have discussed their reviews with one another, and the Reviewing Editor has drafted this to help you prepare a revised submission.

Essential revisions:

1) Reviewer #1 questioned whether the comparison between rate and spike-timing based codes was fair, as the spike-timing code was adjusted for scanning speed, while the rate code was not. Please address this point in the revision; this will likely require additional analysis.

2) Please add a section in the Discussion providing a more in-depth comparison with the psychophysical literature (see comment by reviewer #1).

3) Several reviewers had questions about the stimulus set (choice of stimuli, inclusion of some conditions in the results but not others). Please clarify these in the text.

4) Reviewer #2 asked about the link between the spatial precision of the neurons and the size of a fingerprint ridge. In a somewhat related question, reviewer #1 wondered about the robustness of sub-field responses especially during higher scanning speeds. Please comment on these issues.

*Reviewer #1 (Recommendations for the authors):*

1) In the comparison across different speeds, the temporal profile is corrected for scanning speed, while the spike rates are not. To make a fairer comparison while assuming that scanning speed is accessible, one might fit a linear function to the speed/rate data (see Figure 2) and then 'correct' the rates accordingly. Any systematic deviation in firing rates due to the different orientations should then be discriminable. Given that firing rates do not perform particularly well even in the within-speed discrimination analysis, I would not expect them to outperform the spatial profile in the across-speed analysis either, but such an analysis would provide more robust evidence that rates alone are not sufficient to accomplish fine discrimination.

2) Why was a vertical orientation (0 degrees) not included in the setup? The authors cite 10 degrees as the perceptual lower bound, but this value cannot be directly tested due to the omission of a vertically oriented edge.

3) The comparison with the psychophysical literature could be more extensive. For example, the Bensmaia paper that is cited in the introduction also investigated perceptual thresholds across different scanning speeds, a question that is relevant for the current paper as well. The Peters study tested different edge lengths, which might be compared against the size of the typical receptive field etc.

4) Figure 2: At the highest scanning speeds, the average spike count drops to below 10 spikes. Presumably here not every sub-field does reliably elicit a spike anymore. Are the few spikes that do get elicited randomly distributed across all subfields, or do certain sub-fields reliably produce spikes while others drop out completely? That is, is the signal robust but not strong enough to aid in discrimination, or is the robustness lost?

5) Figure 7 and associated analysis: In the confusion matrices, when orientations are mis-classified, does this appear most often with their closest orientation? Such smoothness would be expected from the interaction of the sub-fields with the differently oriented lines.

*Reviewer #2 (Recommendations for the authors):*

I only have a few comments:

- The data collected at 2.5, 5, and 10 mm/s does not appear in the results, is there a reason?

- The authors should probably elaborate on the link between the spatial precision of the neurons (~60-70 um) and the fingerprint ridges dimension (~300-400um), which I believe are not so close. Also, the edge widths are close to fingerprint ridges periodicity, could this have an artificial effect on the results?

- P16, first paragraph: Delhaye et al., 2019 is a review of primate somatosensory cortex and is not related to the sentence "the viscoelastic and anisotropic properties of the fingertip, and thus possible complex time-varying deformations of the fingertip skin as a surface slides over it".

*Reviewer #3 (Recommendations for the authors):*

This is a very well-written paper. I have no concerns and fully support its publication.

# Reply: Author response

**DOI:** 10.7554/eLife.81476.sa2

Essential revisions:

1) Reviewer #1 questioned whether the comparison between rate and spike-timing based codes was fair, as the spike-timing code was adjusted for scanning speed, while the rate code was not. Please address this point in the revision; this will likely require additional analysis.

2) Please add a section in the Discussion providing a more in-depth comparison with the psychophysical literature (see comment by reviewer #1).

3) Several reviewers had questions about the stimulus set (choice of stimuli, inclusion of some conditions in the results but not others). Please clarify these in the text.

4) Reviewer #2 asked about the link between the spatial precision of the neurons and the size of a fingerprint ridge. In a somewhat related question, reviewer #1 wondered about the robustness of sub-field responses especially during higher scanning speeds. Please comment on these issues.

The above comments have been addressed. See below our response to specific reviewer comments for details.

Reviewer #1 (Recommendations for the authors):

1) In the comparison across different speeds, the temporal profile is corrected for scanning speed, while the spike rates are not. To make a fairer comparison while assuming that scanning speed is accessible, one might fit a linear function to the speed/rate data (see Figure 2) and then 'correct' the rates accordingly. Any systematic deviation in firing rates due to the different orientations should then be discriminable. Given that firing rates do not perform particularly well even in the within-speed discrimination analysis, I would not expect them to outperform the spatial profile in the across-speed analysis either, but such an analysis would provide more robust evidence that rates alone are not sufficient to accomplish fine discrimination.

As the reviewer suggests, the intensity measures could do better discriminating edge orientation across stimulation speeds with some ‘correction’ for stimulation speed. This is the case because, as we show in the paper, stimulation speed very strongly influences the intensity of FA-1 and SA-1 responses. In the limit, with perfect correction for stimulation speed, the neurons would do as well as they do in the within-speed comparison. Performance is already poor within-speed and hence the reviewer rightfully does not expect a correction to yield any qualitative change in interpretation with any correction. Of course, the nervous system cannot perform an ideal correction because it does not have direct access to the speed condition but rather must estimate stimulation speed via neural activity from (some set of) neurons in the population. This leads to the reviewer’s specific suggestion that we apply a correction according to the mean speed sensitivity of the population (as shown in Figure 2). We have done the requested correction which, on average, does not lead to reliable across-speed discrimination performance. The reason that correction by population level intensity information is not particularly effective is because the effect of speed is quite variable across neurons. We have also taken this comment as an opportunity to clarify to the reader why we are doing this analysis. We have made the following changes to the manuscript to help emphasize our analytical intentions and thus clarify the conclusions we believe can be drawn from this study.

“On average, FA-1 and SA-1 neurons carried little information about fine orientation differences even under the assumption that scanning speed is accessible based on the overall intensity of the population response. That is, even after compensating for the effect of scanning speed on a neuron’s response by normalizing its response by a linear function approximating the population level speed-intensity relationship (Figure 2), the neuron’s failed to provide reliable information about edge orientation (peak firing rate: F1,51 = 0.16, p = 0.69, ηp2 = 0.003; mean firing rate: F1,51 = 2.7, p = 0.1, ηp2 = 0.051).

(Results – Speed-invariant orientation signaling)

We are motivated to investigate speed-invariance because speed often varies when touching and manipulating objects but mostly because it provides one means of testing whether the subfield arrangement of FA-1 and SA-1 neurons is an important factor for their signaling of fine differences in edge orientation. (Results – Speed-invariant orientation signaling)

In this scenario, the best-case outcome is that across-speed discrimination performance is equivalent to within-speed discrimination performance for a given speed condition. (Results – Speed-invariant orientation signaling)

As expected, given how strongly intensity measures (i.e., peak and mean firing rates) are affected by the scanning speed (Figure 2), … (Results – Speed-invariant orientation signaling)”

2) Why was a vertical orientation (0 degrees) not included in the setup? The authors cite 10 degrees as the perceptual lower bound, but this value cannot be directly tested due to the omission of a vertically oriented edge.

We omitted a 0° stimulus to efficiently pack stimuli onto the drum which, as the reviewer knows, is critical for microneurography experiments due to the risk of losing isolation. With respect angular differences that can be tested, our present stimuli allow for comparing deltas of 5° degrees (+5° and +10°; -5° and -10° lines), 10° (-5° and +5°), 15° (-5° and +10°; +5° and -10°) and 20° (-10° and +10°). Adding a 0° line stimulus would have added more comparison options for these angular differences but would not yield additional angular differences per se. We now make this logic clear in the text.

“These orientations were chosen to efficiently pack the stimuli onto the stimulating surface and to permit pairwise comparison of lines with angular differences of 5° (between +5° and +10° line stimuli; between -5° and -10° line stimuli), 10° (between -5° and +5° line stimuli), 15° (between -5° and +10° line stimuli; between +5° and -10° line stimuli) and 20° (between -10° and +10° line stimuli). (Methods – Stimuli)

Given our data set, the available angular differences were 5° (between -5° and -10° line stimuli and between +5° and +10° line stimuli), 10° (-5° and +5°), 15° (-10° and +5°; -5° and +10°) and 20° (-10° and +10°). (Methods – Precision of spiking responses)”

3) The comparison with the psychophysical literature could be more extensive. For example, the Bensmaia paper that is cited in the introduction also investigated perceptual thresholds across different scanning speeds, a question that is relevant for the current paper as well. The Peters study tested different edge lengths, which might be compared against the size of the typical receptive field etc.

We agree and appreciate this push. We have now elaborated the relevant text in the Discussion.

“Our findings reveal that single human first-order tactile neurons signal edge orientation most reliably at slow to moderate speeds, corresponding to those used when actively performing tactile spatial discrimination tasks with the fingertips (Olczak et al., 2018; Vega-Bermudez et al., 1991). Our findings also reveal that edge orientation signaling decrease for stimulation speeds exceeding ~45 mm/s, corresponding to the speeds at which spatial discrimination capacity decreases (Bensmaia et al., 2008; Vega-Bermudez et al., 1991). We speculate that the choice of speeds in active tasks and changes in discrimination capacity as a function of speed in passive tasks are associated with the ability of first-order tactile neurons to produce the relevant information. Edge orientation sensitivity at natural speeds was strikingly high, with neurons routinely showing speed-invariant orientation signaling for differences as small as 5°, which is substantially better than what humans can consciously report (Bensmaia et al., 2008; Lechelt, 1992; Olczak et al., 2018; Peters et al., 2015) but on a par with what people exhibit in object manipulation (Pruszynski et al., 2018). (Discussion)

Regarding the conscious perception of edge orientation when edges of different lengths are pressed against a fingertip, humans perform poorly for 2 mm long edges followed by an abrupt improvement for edges longer than 4 mm (Peters et al., 2015). It is tempting to speculate that the substantial performance degradation for 2 mm long edges relates to the fact that, for a vast majority of FA-1 and SA-1 neurons, this length is substantially smaller than the area of the fingertip skin where a neuron's subfields are distributed (Jarocka et al., 2021; Johansson, 1978; Phillips et al., 1992). That is, if the edge length is smaller than the scale of the subfield arrangement, a single neuron likely carries less information about edge orientation compared to edge lengths can stimulate the entire set of subfields. (Discussion)”

4) Figure 2: At the highest scanning speeds, the average spike count drops to below 10 spikes. Presumably here not every sub-field does reliably elicit a spike anymore. Are the few spikes that do get elicited randomly distributed across all subfields, or do certain sub-fields reliably produce spikes while others drop out completely? That is, is the signal robust but not strong enough to aid in discrimination, or is the robustness lost?

The reviewer raises a very interesting question about the nature of subfield spike generation capacity and interactions among subfields – an issue we are presently tackling both empirically and computationally. The present experiment makes it difficult to say anything conclusive, however, since we do not have receptive field maps for these neurons. What is clear is that, over a rather large range, that basic subfield induced structure is maintained (which is why the across speed discrimination functions well for spikes aligned in spatial coordinated) but that this structuring breaks down for very high speeds. A simple (speculative) idea is that, when the speed is very high, the stimulus will traverse a subfield and generate an action potential, and then traverse other subfields while the neuron is still in its refractory period and thus not yield an action potential; in the extreme only one subfield will generate one action potential for all orientations and thus there would be no information about edge orientation in its response pattern. Of course, additional kinds of interactions are possible depending on the biophysics of these neurons, and these could, in principle, open up a host of computational advantages.

5) Figure 7 and associated analysis: In the confusion matrices, when orientations are mis-classified, does this appear most often with their closest orientation? Such smoothness would be expected from the interaction of the sub-fields with the differently oriented lines.

Generally, misclassifications happen to the closest orientations which can be seen in the off-diagonal terms of Figure 7. Note, however, that such smoothness is not an absolute necessity given a neuron’s response profile relates to the geometry of the subfield layout (see Pruszynski et al., *eLife*, 2018). Smoothness can also be seen in Figure 8 in the sense that smaller edge orientation differences are harder to discriminate than bigger edge orientation differences.

“Discrimination accuracy for all combinations of edge orientations and speeds is illustrated as a confusion matrix in Figure 7. Note that the various line stimuli were similarly discriminated and that misclassifications tended to occur towards edges with similar orientation (i.e. off-diagonal terms). (Results – Discrimination accuracy based on convolved spike trains)”

Reviewer #2 (Recommendations for the authors):

I only have a few comments:

- The data collected at 2.5, 5, and 10 mm/s does not appear in the results, is there a reason?

When we started collecting data for this study, we focused on the 15-180 mm/s speeds, which were presented in random order. Later in data collection, we added a 270 mm/s condition and later still, 2.5, 5, 10 mm/s speeds. These slowest three conditions were not randomized and were always collected at the end of data collection because the slow speeds take a very long time and thus we would often lose neuronal isolation before completion. Thus, the core data we have for all neurons is from 15-180 mm/s which gives us power in terms of number and the capacity to treat speed as a repeated measure. We have much lower numbers for the other conditions and thus limits and complicates statistical analysis. That said, these speeds show the expected trends and are included in the openly available dataset attached to this manuscript for future consideration. We now explain this explicitly in the Methods.

“All neurons were scanned at eight tangential speeds: 15, 20, 30, 45, 60, 90, 120, 180 mm/s presented in random order. A subset of neurons was scanned at up to four additional speeds: 2.5, 5, 10, and 270 mm/s. When the 270 mm/s speed was presented, it was randomly interleaved with the main eight speed conditions. When the three slowest speeds were presented, they were always done after all other speed conditions and in a set order, from fastest to slowest (i.e. 10 mm/s, 5 mm/s and then 2.5 mm/s). These very slow speeds take a long time to complete so presenting them at the end minimized the risk of losing isolation of the current neuron during the eight main speeds and thereby creating partial data sets. All the analyses presented here focus on the main speed conditions since these were available for all neurons. Note that the same general trends hold for the additional speeds and that the raw data for all speed conditions is provided alongside this manuscript. For each neuron and speed, the drum rotated three times, resulting in 15 trials for each line stimulus and recorded speed condition (5 presentations of stimuli per drum rotation x 3 drum rotations = 15 trials). (Methods – Stimuli).”

- The authors should probably elaborate on the link between the spatial precision of the neurons (~60-70 um) and the fingerprint ridges dimension (~300-400um), which I believe are not so close. Also, the edge widths are close to fingerprint ridges periodicity, could this have an artificial effect on the results?

Great comment, we agree this was unclear. We have previously dealt with these issues in detail in the context of receptive field maps using small dot stimuli (Jarocka et al., 2021) and the same concepts are in play here. That is, the spatial precision of action potentials reported (~70 um) corresponds to a sinusoid with a spatial period of (~350 um). In terms of the edge width being of similar scale to the ridges, we don’t believe this is likely given the leading edge of the stimulus is likely driving the deflection of ridges (Lamotte and Whitehouse, 1986) which we saw when recording from FA-1 and SA-1 neurons in our previous work in the sense that they responded more to leading than trailing ends of stimuli (Pruszynski and Johansson, 2014). Without fully rehashing the entire discussion from the Jarocka paper, we have added text to make these points and links explicitly.

“Indeed, the spatial precision of elicited action potentials rationally matched spatial acuity of subfield arrangements, which corresponds to a spatial period similar to the dimensions of individual fingertip ridges.” (Abstract)

“Second, in terms of Gaussian kernel widths, the estimate of the spatial precision of action potentials in this study is practically identical to the estimated spatial sensitivity of the subfield arrangement of the FA-1 and SA-1 neurons mapped by small dots laterally scanning the receptive field (Jarocka et al., 2021). For both studies, the estimated spatial sensitivity implies a spatial period expressed in sinusoidal terms that basically matches the width of an individual papillary ridge. That is, based on a Gaussian function being very similar to the period of a cosine cycle specified between -π and π, the spatial sensitivity in Gaussian terms averaged across both neuron types in the two studies corresponds to a sinusoidal period of 0.33 mm and 0.41 mm respectively (for details, see Jarocka et al., 2021). One possibility is that the width of the line stimuli could have acted as a low-pass filter and thus led to an underestimation of spatial sensitivity. However, we do not believe this to be the case given previous work showing that ridge deflections that stimulate FA-1 and SA-1 neurons are largely driven by the leading edge of similar stimuli and that the neurons, as well as their monkey equivalents, generally respond more intensely to the leading rather than trailing ends of tactile elements (Blake et al., 1997; LaMotte & Whitehouse, 1986; Pruszynski & Johansson, 2014).” (Discussion)

- P16, first paragraph: Delhaye et al., 2019 is a review of primate somatosensory cortex and is not related to the sentence "the viscoelastic and anisotropic properties of the fingertip, and thus possible complex time-varying deformations of the fingertip skin as a surface slides over it".

Our apologies, that was a typo, we have now corrected this citation to point to Delhaye et al, 2016 (https://doi.org/10.1098/rsif.2015.0874).

“Second, our analysis ignored the viscoelastic and anisotropic properties of the fingertip, and thus possible complex time-varying deformations of the fingertip skin as a surface slides over it (Delhaye et al., 2016; Jarocka et al., 2021).” (Discussion)

Reviewer #3 (Recommendations for the authors):

This is a very well-written paper. I have no concerns and fully support its publication.

We appreciate the positive feedback.
